# Nurses and Pharmaceutical Care: Interprofessional, Evidence-Based Working to Improve Patient Care and Outcomes

**DOI:** 10.3390/ijerph18115973

**Published:** 2021-06-02

**Authors:** Tinne Dilles, Jana Heczkova, Styliani Tziaferi, Ann Karin Helgesen, Vigdis Abrahamsen Grøndahl, Bart Van Rompaey, Carolien G. Sino, Sue Jordan

**Affiliations:** 1 Centre for Research and Innovation in Care (CRIC), Nurse and Pharmaceutical Care (NuPhaC), Department of Nursing and Midwifery Science, Faculty of Medicine and Health Sciences, University of Antwerp, 2610 Antwerp, Belgium; bart.vanrompaey@uantwerpen.be; 2 First Faculty of Medicine, Institute of Nursing Theory and Practice, Charles University, 11000 Prague, Czech Republic; jana.heczkova@lf1.cuni.cz; 3 Laboratory of Integrated Health Care, Department of Nursing, University of Peloponnese, 22100 Tripolis, Greece; stylianitziaferi216@gmail.com; 4 Faculty of Health and Welfare, Østfold University College, 1757 Halden, Norway; ann.k.helgesen@hiof.no (A.K.H.); vigdis.a.grondahl@hiof.no (V.A.G.); 5 Research Group Care for the Chronically Ill, University of Applied Sciences Utrecht, 3584 CH Utrecht, The Netherlands; carolien.sino@hu.nl; 6 Department of Nursing, Swansea University, Swansea SA2 8PP, Wales, UK; s.e.jordan@swansea.ac.uk

**Keywords:** nursing, pharmaceutical care, interprofessional collaboration

## Abstract

Pharmaceutical care necessitates significant efforts from patients, informal caregivers, the interprofessional team of health care professionals and health care system administrators. Collaboration, mutual respect and agreement amongst all stakeholders regarding responsibilities throughout the complex process of pharmaceutical care is needed before patients can take full advantage of modern medicine. Based on the literature and policy documents, in this position paper, we reflect on opportunities for integrated evidence-based pharmaceutical care to improve care quality and patient outcomes from a nursing perspective. Despite the consensus that interprofessional collaboration is essential, in clinical practice, research, education and policy-making challenges are often not addressed interprofessionally. This paper concludes with specific advises to move towards the implementation of more interprofessional, evidence-based pharmaceutical care.

## 1. Introduction

Prescribed and purchased medicines are important aspects of patient management. Optimising and individualising each patient’s pharmacotherapy regimen, with maximum therapeutic gain and minimum adverse effects, can be challenging. Pharmaceutical care, with the focus on optimising medicine use and the improvement of health outcomes [1], necessitates significant efforts from patients, informal caregivers, the interprofessional team of health care professionals and health care system administrators. Collaboration, mutual respect and agreement amongst all stakeholders regarding responsibilities throughout the complex process of pharmaceutical care is needed before patients can take full advantage of modern medicine.

On 11 March 2020, the Council of Europe adopted a new resolution on pharmaceutical care [2]. Pharmaceutical care was defined as the responsible provision of pharmacotherapy for the purpose of achieving definite outcomes that improve a patient’s quality of life [2,3]. Examples of definite outcomes reported in core outcome sets are drug-related hospital admissions, drug overuse, drug underuse, potentially inappropriate medications/medication appropriateness, clinically significant drug–drug interactions, health-related quality of life, pain relief, adverse drug reactions, falls, medication regimen complexity, mortality, and medication side effects [4,5]. The resolution focuses on how pharmaceutical care can be implemented for the benefit of patients and health services. Patients and their families or friends are not only important partners in care, they also decide on care goals, informed by health care providers. They are key in the evaluation of care and the achievement of anticipated care goals.

The resolution identifies opportunities to optimise pharmaceutical care through interprofessional and patient-centred approaches, but also some challenges. Steps in pharmaceutical care, listed in the resolution, are (1) patient assessment of medication, health problems and health status; (2) identification and prioritisation of medication-related problems; (3) selection of interventions and formulation of pharmaceutical care plan; (4) patient agreement, implementation and monitoring; and (5) follow-up with the patient [2]. Other concepts are sometimes used to refer to (parts of) pharmaceutical care as defined above: one example is the concept of medicines optimisation, as defined by the UK National Health Service (NHS) [6]. This paper embraces these concepts to the extent that they accord with the definition of pharmaceutical care.

The resolution acknowledges the importance of an integrated interprofessional and multi-disciplinary approach to improving quality of care and patient outcomes. The World Health Organization (WHO) defines integrated health services as “health services that are managed and delivered so that people receive a continuum of health promotion, disease prevention, diagnosis, treatment, disease-management, rehabilitation and palliative care services, coordinated across the different levels and sites of care within and beyond the health sector, and according to their needs throughout the life course” [7]. Person- or people-centred care is a prerequisite for integrated care. In an editorial, J. Scerri et al. explain that person-centred care can impact the regulatory and decision-making context for the safe use of medicines at the clinical level [8]. Person- or people-centred care entails goal-oriented care, with a focus on the person instead of on the patient or the disease: it can be delivered in the absence of disease. It promotes equality in the relationship between health care providers and patients. This framework explores the needs and expectations of the person, considering the context of the patient, family and community. People-centred care aims to provide the education and support for individuals to make decisions and participate in their own care [7,9]. So, by definition, people-centred pharmaceutical care requires regular communication between patients and health care providers, patient education, monitoring and tailoring of care and interventions. Medication use needs to be adjusted in accordance with patients’ care goals and contextual factors such as patient competences, therapy expectations, financial means, informal care and beliefs about medication.

Integrated care requires intense interprofessional collaboration. To implement high-quality interprofessional relationships in pharmaceutical care, health care providers need to acknowledge shared, person-centred goals and respect each other’s competences and contributions.

Frameworks are needed for co-operation and communication and for establishing trust [2]. In this position paper, we reflect on opportunities for integrated evidence-based pharmaceutical care to improve care quality and patient outcomes from a nursing perspective. While the resolution mainly focuses on what would be needed from the viewpoint of pharmacy services, we want to raise some points from the viewpoint of nurses. Nurses have an important role in interprofessional pharmaceutical care. When building implementation frameworks for pharmaceutical care, policy makers and managers need to consider not only what health care providers can or want to do but also feasibility, equity and cost-effectiveness, always with the focus on care quality and patient outcomes.

## 2. Methods

Based on the literature and policy documents, in this position paper, we reflect on opportunities for integrated evidence-based pharmaceutical care to improve care quality and patient outcomes from a nursing perspective.

## 3. Results

### 3.1. Nurses’ Contribution to Interprofessional Pharmaceutical Care

The WHO explains on its website that “nursing encompasses autonomous and collaborative care of individuals of all ages, families, groups and communities, sick or well, and in all settings. It includes the promotion of health, the prevention of illness, and the care of ill, disabled and dying people. Nurses play a critical role in health care and are often the unsung heroes in health care facilities and emergency responses. They are often the first to detect health emergencies and work in the front lines of disease prevention and the delivery of primary health care, including promotion, prevention, treatment and rehabilitation” [10,11].

Nurses contribute to pharmaceutical care on a daily basis. In line with WHO statements regarding nursing and health care more generally, in pharmaceutical care, nurses provide autonomous and collaborative care in the front lines, including health promotion, illness prevention, treatment and rehabilitation, for most populations. They closely support patients in managing their medicines, monitoring effects and any adverse side effects of medicines and preventing drug-related problems, for example, by checking medicines before administration [12,13,14,15]. A positive impact on care quality is associated with nurses assuming responsibilities in pharmaceutical care. In nursing homes and community care, nurses’ observations have significantly improved the detection of drug-related problems and the number of drug-related problems addressed [16,17,18,19]. Using Pharmanurse software, nurses observed a total of 821 adverse drug reactions, confirmed by physicians, in 60% of 418 nursing home residents. The observations directly resulted in 214 medication changes to address patients’ problems [18]. After integration of the software in a multidisciplinary platform to optimise medication use (OptiMEDs), the use of potentially inappropriate medications decreased in 26% of residents [20]. Descriptions of nurses’ interventions in outpatient consultations demonstrated nurses’ contributions to effectiveness, safety and efficiency in pharmaceutical care, for example, by the detection of discrepancies in (para)medical records on treatments, allergies or intolerances or by patient education on discharge [21]. Doctors and pharmacists expect nurses to make observations and assessments of key patient information, to be shared and addressed by the interprofessional team [12,22].

Nurses are members of interprofessional teams, acting in multiple roles: caring and advocating for patients; supporting and educating them on the path from diagnosis to treatment and evaluation; and implementing health care interventions [23,24]. Across Europe, different levels of nurse education are embedded in different legal contexts. Responsibilities range from medicine administration to prescribing. Pharmaceutical care rarely stands alone, and it is embedded in multidisciplinary treatment. In accordance with their positions and roles in the multidisciplinary team, nurses play a crucial role in ensuring patient safety in pharmaceutical care [25,26,27,28,29,30]. Transition to person- and people-centred care increases demands on health care providers, who need to invest in regular communication with patients, patient education and monitoring and tailoring of care. Patients receiving nursing care are generally more often in contact with their nurses than with other health care providers. Nurses have the competences and the opportunities to be the eyes and the ears of the multidisciplinary team, to interact with patients, to learn and understand patients’ needs and expectations, to monitor the effects of therapeutic regimens and to share these findings in an interprofessional team. This way, they not only improve nursing care but also provide data for physicians and pharmacists to improve medical and pharmacist care. Hence, aspects of the pharmaceutical care such as interdisciplinary communication, supporting of patients during the medication process and follow-up for benefit or harm should be shared with nurses to consolidate their roles as key pharmaceutical care providers [31].

### 3.2. Acknowledging the Roles of Nurses in Pharmaceutical Care

As described in the resolution of the Council of Europe on the implementation of pharmaceutical care, full recognition of nurses’ roles is essential [2,32]. Nurses play a central role in pharmaceutical care and, as those administering medicines, constitute the last link of the patient safety chain [33,34]. Nevertheless, nurses’ roles are not always acknowledged. One definition from Pharmaceutical Care Network Europe claimed pharmaceutical care as pharmacist care. This was decided in the last round of a pharmacist-only expert meeting. Results of previous rounds of the expert meetings show differences in opinions as to who can provide pharmaceutical care [1]. Nurses’ roles in pharmaceutical care are not always clear, which increases the risk for these roles to be minimised or even ignored [12,13]. Interviews with pharmacists, physicians and nurses in 14 European countries illustrate that while many report examples of valuable nurse contributions and the strength of interprofessional teamwork, others do not acknowledge these contributions [35].

Only by fully acknowledging nurses’ roles can health care systems invest in upskilling nurses through research, nurse education and policy decisions. Successful implementation of integrated evidence-based pharmaceutical care requires a strong team approach and interprofessional collaboration, not only in clinical practice, but also in research, education and policy making.

Recognition of nurses’ roles will allow nurses to collaborate more explicitly in research and policy making [36]. The literature on interventions to improve pharmaceutical care shows these interventions sometimes lack an interprofessional approach and seldom incorporate nurses’ contribution to pharmaceutical care, beyond the administration of medication. In addition, nurses are seldom investigators in the research teams. In a Cochrane review on interventions for improving medication-taking ability and adherence in older adults prescribed multiple medicines, in 15/50 studies interventions were delivered in teams of more than one profession (physician or pharmacist or nurses together) and in 17/50 studies nurses were involved [37]. A Cochrane review of medication review in hospitalised patients reported no nurse involvement in the interventions included [38]. In these innovative interventions, opportunities to organise efficient care with optimal patient outcomes through interprofessional collaboration seem to have been overlooked. In addition, when plans are made, decisions on nursing care are often made by other disciplines. Accordingly, decisions may not fully reflect nurses’ professionalism, experiential learning and daily experience and may unknowingly overlook elements essential for successful implementation. In times of shortage of health care professionals, high workloads and restricted budgets, this is unacceptable. Implementation frameworks should consider all the disciplines involved and the different levels of expertise within each discipline, such as the EU levels of nursing competence [35,39]. Assigning responsibilities and tasks to disciplines or levels should be aligned to their competences, availability and costs, while maximising care quality and patient safety. Evidence suggests that nurses can contribute very effectively health care provision by assuming responsibilities traditionally not seen as nursing tasks and are able to provide advanced care, including prescribing, at equal (or even higher) standards than other health care professionals [40,41]. Role extension should never be a goal in itself but should gain acceptance if it promotes efficient and effective pharmaceutical care. Interprofessional implementation frameworks for disciplines and levels to inter-relate to enhance care quality and patient outcomes should underpin future quality improvement projects in pharmaceutical care. Full acknowledgement of nurses’ roles will facilitate the development of interprofessional and multidisciplinary integrated care plans, focusing on person-centred care, quality of care and patient outcomes [42]. Full recognition of nurses’ roles would facilitate research investment into the juxtaposition of nursing care with pharmaceutical care, and progress the patient safety agenda [43]. This paper offers some examples:

- Nurses or carers monitoring patients for possible adverse effects of prescribed medicines improve patient outcomes, including reduced pain, sedation, dyspnoea, aggression and infections. In addition, contacts with dentists, opticians and primary care doctors increase [44,45,46].
- Nurses following-up with caregivers to initiate medication reconciliation lowers 30-day hospital readmission rates [47].
- Nurses conducting initial assessments, including extensive medication reviews, in collaboration with pharmacists, provide accurate discharge medication charts, adjust medications and order medication renewals. As a result, hospital admissions fell by 67% and emergency department visits fell by 61% in the 90 days after participation in the program [48].

Acknowledging nurses’ roles is not only important to research and policy making, it is also essential to nurse education. Nurses are expected to be fully competent in pharmaceutical care to fulfil their roles (including those in pharmaceutical care) at the point of graduation. However, demands of current practice sometimes greatly exceed expectations that can be derived from formal requirements of nurses’ competencies in education programmes [49]. Unless nurses’ roles in pharmaceutical care are recognised, nursing curricula cannot prepare nursing students for their roles in clinical practice. Nursing curricula, at undergraduate and postgraduate levels, have to be based on structures, processes and outcomes that lead to skills and knowledge that benefit clinical practice [50]. This particularly applies to pharmaceutical care, as a crucial element of nursing care.

### 3.3. Nurse Education in Pharmaceutical Care

Nursing undergraduate programs throughout Europe offer students theoretical backgrounds and practical training in general terms, including administration of medicines. Teaching and learning methods usually include simulation exercises and online learning modules. These often incorporate medication administration safety, and sometimes they address prevention of medication errors, but rarely prescribing, and patient follow-up for adverse side effects. This educational background allows nurses to develop medication administration skills in safe educational environments, without the distraction of the real clinical world [51,52].

The literature indicates shortcomings in nurses’ competences in pharmaceutical care [53]. Such deficits are also reported amongst other health care professionals, including midwives, doctors and pharmacists [54,55,56,57,58,59,60,61]. Shared interdisciplinary courses for all health care professionals in topics such as pharmaceutical care can allow students to experience opportunities for collaboration, with shared goals and action plans for enhanced patient safety [62]. Accordingly, a clear framework for health care providers’ shared roles in pharmaceutical care is needed to create opportunities to strengthen educational preparation. The first models of nurses’ responsibilities and tasks in pharmaceutical care, as developed in the European DeMoPhaC project, offer guidance [12,39].

### 3.4. Towards Integrated Evidence-Based Pharmaceutical Care

A collaborative environment entails sharing. Shared responsibilities, goals and identity represent core elements of interprofessional practice [63]. However, interprofessional collaboration is vulnerable to barriers engendered by socio-cultural contexts and organisational hierarchies, which impact attitudes towards other people or professional groups [64]. However, interprofessional collaboration has potential to address serious health care safety issues [65] and improve job satisfaction [66]. Interprofessional collaboration is more than multidisciplinary task management [67]. Effective collaboration requires sharing goals, sharing some responsibilities, sharing some tasks [68]. In contrast, fighting over roles and responsibilities with the aim of defending or extending professional identities and territories hinders collaboration. Lack of collaboration between professionals results in unsatisfactory, suboptimal care, delays in provision of care, more clinical errors, demotivation of professionals, negative attitudes towards patients and low patient satisfaction [69,70].

In teams with strong collaborations, responsibilities and tasks overlap amongst professional groups, with professionals working together to achieve clinical goals [63]. Extra effort to move beyond profession-specific competencies will be necessary for enhanced collaboration, team-based care and cross-disciplinary working in order to improve health outcomes [71]. Trust, mutual respect and shared values within interprofessional teams have to be seen as fundamental prerequisites [71,72] to maximise the potential contribution of each professional group to achieve patient safety.

Emphasis on the surrogacy of roles and the transmission of responsibilities contributes to the effectiveness and productivity of services [73]. According to the Νursing Now campaign, “Changing needs of the 21st Century mean nurses have an even greater role to play in the future. New and innovative types of services are needed—more community and home-based, more holistic and people-centered, with increased focus on prevention and making better use of technology. These are all areas where nurses can play a leading role. However, maximizing nurses’ contributions will require that they are properly deployed, valued and included in policy and decision-making” [74]. Approaching pharmaceutical care as an interprofessional advanced practice, with central roles for pharmacists and physicians yet with nurses as vital team members, will contribute to the quality of nurse education, pharmaceutical care research, the quality of pharmaceutical care delivered by nurses, safe medication management, optimum patient outcomes, job satisfaction and efficient use of health care budgets and staff [72].

## 4. Advices to Strengthen Integrated, Evidence-Based Pharmaceutical Care

**Figure 1:**
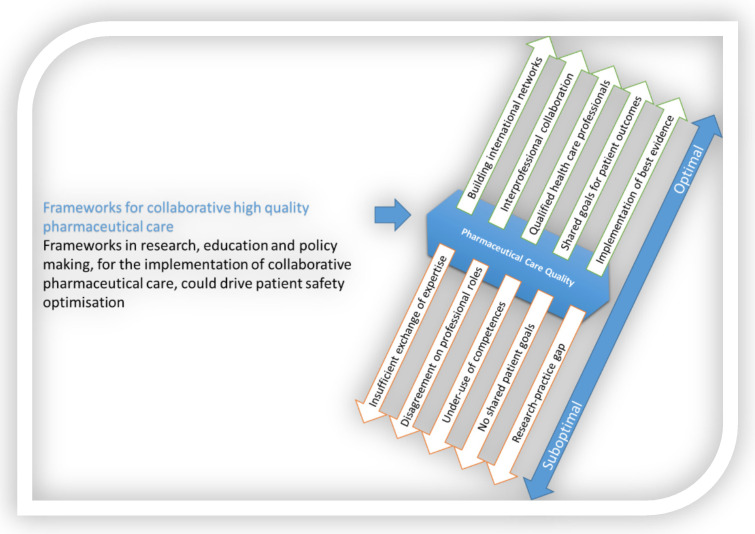
The need for frameworks to allow the implementation of interprofessional, integrated, evidence-based pharmaceutical care.

Many factors impact the quality of integrated, evidence-based pharmaceutical care, as schematically presented in Figure 1. Measures to strengthen integrated, evidence-based pharmaceutical care can address these factors. To support implementation, nurse leaders could and probably should or even must:

- **Offer a framework for nurses’ contributions to integrated pharmaceutical care.**
  Nurses need to communicate clearly on how they can and do contribute to integrated pharmaceutical care.
- **Expand research on nursing interventions in pharmaceutical care.**
  Nurses need to extend the available evidence on how they can contribute to improvement of clinical practice [75,76,77].
- **Expand research on interprofessional collaboration in pharmaceutical care.**
  As interprofessional collaboration in pharmaceutical is so fundamental to the quality of integrated, people-centred care and patient outcomes, research should continue to focus on facilitators of high-quality interprofessional pharmaceutical care [78] and the barriers to bringing this into practice. This research should also consider how gender, gender-based assumptions, stereotypes and preconceptions still influence participants [79].
- **Ensure that available evidence is implemented in practice.**
  Substantial parts of evidence resulting from research are not translated into practice and therefore fail to generate expected outcomes. Therefore, extra efforts from all professionals involved (clinicians, managers, regulators and policy makers) are needed to ensure research results are implemented in clinical practice [80].
- **Create and maintain an international network of nurses with expertise in pharmaceutical care.**
  Such a network serves not only to enhance collaboration, exchange initiatives and disseminate information but also to provide a point of contact for other professional groups to identify nurse representatives to be engaged in research and policy making on pharmaceutical care. Engaging in debates at this level will develop interprofessional frameworks for the implementation of pharmaceutical care and strengthen nurses’ contribution to research, policy, education and clinical practice.
- **Collaborate with representatives of other disciplines to develop a framework for interprofessional pharmaceutical care.**
  The framework should be developed and co-designed as a collaboration between all disciplines involved in pharmaceutical care. The goals are optimum care quality and patient outcomes, allowing for contextual factors, such as expertise, treatment availability and costs. Knowledge gained from implementation science models, such as the CICI framework, can be harnessed to improve the usability of such frameworks [81] and uncover and address any challenges in implementing the framework.

Guidelines and policy steers should be provided so that nurses could, and probably should, in line with international priorities, contribute to:

- **Ensuring best use of medicines**
  Nurses, as the professionals working most closely with patients, should be fully integrated into the interprofessional team providing pharmaceutical care, including managing and monitoring patients’ medicines and the effects of these medicines on patients [16,18,31,46].
- **Eliminating inequity**
  The inequity in the outcomes and processes of care is often based on socio-economic or territorial inequalities, resulting in restricted access to quality health care services. Nurses have potential to contribute to pharmaceutical care and to deliver safe and optimal pharmaceutical care [14,82]. There is little evidence that nurse substitutions [83] or nurse practitioners’ care [76,84] or costs [85] differ from those of doctors, particularly when prescribing practices are compared. The evidence suggests that non-medical prescribing is safe and can provide beneficial outcomes [40], even though nurses tend to prescribe less than doctors [86] and have reservations regarding working unsupported [12,76,86].
  Furthermore, nurses, together with pharmacists and physicians, need to explicitly take into account the impact of social diversity when researching and developing interventions or guidelines or policies or providing education in pharmaceutical care. Interprofessional collaboration with shared goals on the elimination of inequality in pharmaceutical care can help us move forward.
- **Promoting patient safety**
  Patient safety related to pharmaceutical care is suboptimal [43]. The adoption of standardised international pharmacotherapy curricula and assessments for pre- and post-registration nurse education would provide the foundation for nurses to meet practice requirements and realise their full potential, whilst maintaining comparable standards of care, not only at national levels, but also at European and international levels [87,88]. Avoiding unsafe medication practices, minimising avoidable harm caused by medicines and meeting the WHO third patient safety challenge [43] requires a focus on patient outcomes.

## 5. Conclusions

Initiatives have been taken to work on the implementation of integrated evidence-based pharmaceutical care. In 2015, the NuPhaC network was founded as a European collaboration to unite researchers, clinicians, educators and policy makers in promoting the quality of pharmaceutical care and patient outcomes by realising the potential of all professionals.

This paper reflected on policy documents to formulate advices to move towards more interprofessional, integrated, evidence-based pharmaceutical care. We hope this paper can convince and motivate health care providers and their representatives to seize the opportunities to go for it together, with a shared focus on what is best for the patient.
